# Epidemiology and Risk Assessment of Pediatric Venous Thromboembolism

**DOI:** 10.3389/fped.2017.00068

**Published:** 2017-04-10

**Authors:** Arash Mahajerin, Stacy E. Croteau

**Affiliations:** ^1^CHOC Children’s Specialists, Orange, FL, USA; ^2^Boston Children’s Hospital, Boston, MA, USA

**Keywords:** epidemiology, pediatrics, thrombosis, risk factors, risk assessment

## Abstract

The incidence of diagnosed venous thromboembolism (VTE) has been increasing concurrent with advances in technology and medical care that enhance our ability to treat pediatric patients with critical illness or complex multiorgan system dysfunction. Although the overall incidence of VTE is estimated at 0.07–0.49 per 10,000 children, higher rates are observed in specific populations including hospitalized children, those with central venous catheters (CVCs) or patients convalescing from a major surgery. While the absolute number of pediatric VTE events may seem trivial compared to adults, the increasing incidence, associated with increased mortality and morbidity, the availability of novel therapies, and the impact on the cost of care have made investigation of VTE risk factors and prevention strategies a high priority. Many putative risk factors for pediatric VTE have been reported, primarily from single-institution, retrospective studies which lack appropriate methods for verifying independent risk factors. In addition, some risk factors have inconsistent definitions, which vex meta-analyses. CVCs are the most prevalent risk factors but have not consistently been assigned the highest level of risk as defined by odds ratios from retrospective, case–control studies. Few risk-assessment models for hospital-acquired pediatric VTE have been published. Some models focus exclusively on hospitalized pediatric patients, while others target specific populations such as patients with cancer or severe trauma. Multicenter, prospective studies are needed to identify and confirm risk factors in order to create a pediatric risk-assessment tool and optimize preventive measures and reduce unintended harm.

## Introduction

Understanding and intervening on preventable factors that provoke venous thromboembolism (VTE) in pediatrics is a leading initiative for children’s hospitals (1). As the second leading cause of hospital-acquired morbidity (preventable harm) for children in the U.S., VTE significantly increases hospitalization costs. Using the nationwide inpatient sample, one recent analysis estimated increased mean hospital costs of $27,686 and mean length of stay (LOS) extension of 8.1 days in children with hospital-acquired VTE (HA-VTE) compared to controls (2).

The foundation of our current knowledge of pediatric VTE has emerged primarily through registries, administrative databases, and retrospective cohort studies (3–8). Differences in the patient population included and analysis make comparison of these studies challenging. The relative contributions of genetic, anatomic, and acquired risk factors in pediatrics have been less studied than in adults (9, 10). Sequelae of VTE, namely post-thrombotic syndrome (PTS) and recurrence risk, have not been fully investigated in children, but efforts to standardize outcome measures are underway (11–13). This pediatric VTE compendium contains mini-reviews on VTE in the setting of cancer, congenital heart disease, inflammatory states, central venous catheters (CVCs), trauma, and thrombophilia. This mini-review will focus on epidemiology of pediatric VTE as well as patient and acquired risks. For additional discussion of pediatric VTE in a specific disease or clinical state, see the accompanying mini-reviews.

## Incidence

The estimated incidence of pediatric VTE in developed countries ranges from 0.07 to 0.49 per 10,000 children (3, 14). VTE rates are notably higher in hospitalized children, 4.9–21.9 per 10,000 hospital admissions (3, 14, 15). A bimodal distribution is evident. The most prominent peak is in early infancy accounting for up to 20% of pediatric VTE. A second peak occurs during adolescence with about 50% of VTE events occurring in children 11–18 years old. Reported incidence rates vary due to differences in study design, cohort inclusion criteria (e.g., all events versus symptomatic events, whether neonates are included, and/or if age-specific sub-analyses are performed), whether the source of information is a database based on billing codes or whether radiological confirmation was required for inclusion. For example, VTE incidence rates in the Netherlands decreased from 0.14 per 10,000 children to 0.05 per 10,000 children when neonates and non-extremity VTE events were excluded (8).

While the annual burden of thrombosis is greater in adults (5.6–16 per 10,000 adults per year) (9, 16), the rate of increase in observed incidence in children discharged from tertiary care hospitals is notable. Cohort studies targeting HA-VTE demonstrate a steep rise in incidence, increasing from 0.3 to 28.8 cases per 10,000 admissions (1992–2005) in one study and from 34 to 58 cases per 10,000 admissions (2001–2007) in another (17, 18). In contrast to the majority of studies focused on hospital-based populations, longitudinal data from a population-based cohort study in Québec, QC, Canada found a stable VTE incidence of 0.29 events per 10,000 person-years in children less than 18 years old over the 11-year study period ending in 2004 (5). Notably, this study excluded patients less than 1 year of age, and rates were calculated based on person-years, as opposed to VTE rate per hospital admissions.

The majority of VTE diagnosed in children arise proximal to hospitalization and are considered *provoked*. At least one risk factor is identified in the majority of patients (19–21). This stands in contrast to adults where 30–50% of VTE events are idiopathic or spontaneous. CVC-associated VTE predominates in pediatrics. In the absence of a CVC, however, location frequency of VTE varies by age group. Renal vein VTE comprises a significant proportion of events in neonates but is exceedingly rare in older children where lower extremity VTE is more likely. Although rare, non-extremity VTE in children may arise in portal, splenic, mesenteric, pulmonary vessels, or cerebral sinuses (22). No racial or ethnic variations have been described, and data on gender differences are conflicting.

Pediatric subpopulations have higher VTE incidence including children with critical illness, neoplasm, renal disease (nephrotic syndrome), congenital heart disease, inflammatory bowel disease, or obesity and neonates. Children admitted to the intensive care unit (ICU) have a 2% higher risk of VTE if they had a short-term CVC and LOS greater than 7 days (23). Occurrence of VTE in neonates has been estimated as high as 24 per 10,000 neonatal intensive care admissions (20). There are presently no standard thromboprophylaxis guidelines to mitigate increased VTE risk.

## Risk Factors

Many patient attributes, medical diagnoses, and elements of hospitalized care have been shown or suggested to confer HA-VTE risk. Risk factor data are predominately derived from single-institution, retrospective cohort, or case–control studies (10).

### Immobility

Absence of consensus on terms *altered mobility* versus *immobility* creates a challenge when attempting to define the associated risk of these states and potential risk reduction with intervention. Data from adults show a strong association with various etiologies of immobility, e.g., post-surgery, plaster cast. Altered mobility without immobilization also increases risk of symptomatic DVT but to a lesser extent than chronic immobilization (24, 25). Wells’ criteria for suspected DVT have “recently bedridden for 3 days or more” in their clinical model (26). The benchmark of 3 days or more has previously been suggested to confer a high degree of risk in pediatrics but has not been prospectively studied (27). Multiple studies have implicated altered mobility as a risk factor for pediatric HA-VTE. Unfortunately, the granularity to understand and compare duration or degree of altered mobility among studies is lacking (10).

The Braden Q mobility assessment offers a guide for grading mobility (28); however, HA-VTE risk has not been clinically correlated to a threshold score. Prospective evaluation of mobility rubrics in both children and adults is needed to determine VTE risk associated with different mobility states.

### Infection

Infection is often cited as a risk factor without descriptors of location, extent, severity, or inciting organism (29, 30). Occasionally, a specified set of infectious conditions (e.g., meningitis, bacteremia) are categorized as “infection” (31), or infection is dichotomized to focal or systemic. Systemic infections are thought to confer higher risk than a focal infection, but this may depend on location. For example, otitis media or mastoiditis are considered “focal” infections and associated with increased risk of cerebral sinus venous thrombosis (32). Similarly, acute osteomyelitis has been noted to increase risk of VTE in adjacent veins (33). Evaluation of different infectious events and reporting confirmation, e.g., culture, is needed to understand VTE risk of focal and systemic infections. Concomitant inflammation likely plays a role but may prove difficult to identify independent risk separate from the infection.

### Intensive Care Unit

Recent work has evaluated whether admission to or prolonged stay in the ICU is a risk factor for HA-VTE. One study demonstrated ICU admission confers independent VTE risk in all pediatric patients (27). Two studies in pediatric trauma have demonstrated independent VTE risk with ICU admission and ICU stay ≥4 days (34, 35). It is likely that ICU admission or prolonged stay is a proxy for illness severity and need for additional interventions, e.g., CVCs, mechanical ventilation that directly contributes to increased VTE risk.

### Length of Stay

While recent data have highlighted extended LOS *following* HA-VTE, the mechanism by which prolonged hospitalization increases risk is less clear (2). Compounding the challenge are differences in analysis. LOS is a continuous variable but has been analyzed as both a continuous and a dichotomous variable. In two independent retrospective, case–control studies, Branchford et al. and Sharathkumar et al. utilized greater than 5 and 7 days, respectively, as cutoff values for increased VTE risk based on the distribution around the day of hospitalization on which VTE occurred (27, 36). Branchford et al. reported the odds of HA-VTE increasing by 3% for each additional day beyond 5 days. Pediatric trauma-specific literature has demonstrated daily increases in HA-VTE risk of 2 and 3% for those admitted with traumatic brain injury and general trauma, respectively (37, 38). Future research detailing increases in daily risk in the context of concomitant risk factors and whether risk can also decrease with elimination of other risk factors is lacking but would prove beneficial in understanding how LOS impacts VTE risk.

### Mechanical Ventilation

Mechanical ventilation is emerging as a risk factor, but the magnitude of independent risk is unclear. Similar to ICU admission, mechanical ventilation may be a proxy for a severely ill child. In both trauma and critical care pediatric populations, mechanical ventilation has been identified as an independent VTE risk factor (36–38). One study observed an increased risk with ≥4 days of mechanical ventilation (35). Determining how independent risk for VTE increases with each additional day of mechanical ventilation, outside of the trauma setting requires investigation.

### Obesity

Being overweight and obese in pediatrics is defined as body mass indices of 85th–94th and ≥95th percentiles, respectively (39). Obesity increases VTE risk through a chronic low-grade inflammatory state, platelet activation, and endothelial dysfunction (40). VTE risk due to obesity has been well-characterized in adults (41). Minimal pediatric-specific data exist. A retrospective, case–control study of 48 children with VTE identified increased risk in obese children but not overweight children. This study was confounded by frequent co-occurrence of known risk factors in obese children and a small sample size (42).

### Oral Contraceptive Pills

Venous thromboembolism risk with combined oral contraceptive pills (COCPs) has been studied extensively. Estrogen has a multitude of mechanisms that increase thrombotic risk including increases in pro-coagulant proteins, decreases in counter-regulatory proteins including protein S and antithrombin, and inducing protein C resistance (43). While differences exist between route of delivery, type of progesterone, and the doses of estrogen and progesterone, the highest level of risk is thought to occur in the first 3 months of use and gradually plateaus after 12 months of use (44). The overall relative risk is threefold to fivefold higher than non-users of COCPs. Risk increases significantly with concomitant inherited thrombophilia (45). Studies examining VTE risk of progesterone-only contraception are conflicting. Some studies have shown increased risk, particularly with depot medroxyprogesterone and high-dose oral progesterone, whereas other studies have observed no increase in baseline risk, primarily with low-dose oral progesterone and progesterone-only intra-uterine implantable devices (44).

### Surgery

Similar to infection, analyzing surgery is problematic given broad use of the term. Surgery is often reported as a risk factor without defining risk related to a specific procedure or its duration (15, 46). Procedures may be divided into major or minor, but these are neither consistently nor uniformly defined. For example, Van Arendonk et al. defined major surgery as involving the nervous, respiratory, cardiovascular, digestive, urinary, or musculoskeletal systems or spleen (38). Furthermore, not all studies defined the time interval between surgery and VTE. Previous work has defined VTE risk as 7 and 15 days prior to VTE diagnosis (46, 47). The duration of surgery has yet to be explored in pediatrics, but adult data have shown increasing VTE risk with increasing surgery duration (48).

## Risk-Assessment Models

Several risk-assessment models have been published for pediatric HA-VTE (Table 1) (23, 36, 49–52). Branchford et al. showed independent risk with mechanical ventilation, systemic infection, and hospital stay ≥5 days, and that these three factors co-occurring yielded a posttest probability of 3.1% for HA-VTE (36). Sharathkumar et al. derived six independent risk factors and assigned points from beta coefficients in the logistic regression model: immobilization (3), LOS ≥ 7 days (2), OCPs (2), CVC (1), bacteremia (1), and direct ICU admission (0.5). A cumulative score of ≥3 yielded a positive predictive value of 2.45% for HA-VTE at a prevalence of 0.71% (27).

**Table 1:** **Pediatric venous thromboembolism risk-assessment models**.

	Branchford et al. (36)	Sharathkumar et al. (27)	Arlikar et al. (23)	Atchison et al. (49)	Reiter et al. (52)^a^	Kerlin et al. (50, 53)
Pediatric population	All	All	ICU	Non-ICU	ICU	All
Study design for score derivation	Retrospective case–control (1:2)	Retrospective case–control (1:2)	Retrospective case–control (1:3)	Retrospective case–control (1:7)	Literature review	Retrospective cohort
*N*	78:160	173:346	57:171	50:350		389
Validation method		Retrospective case–control (1:1)			Prospective, observational cohort study	Retrospective cohort
*N*		100:100			742	149
Risk factors comprising score	MV Infection LOS ≥ 5 days	Immobilization	CVC LOS ≥ 4 days Infection	CVC Infection LOS ≥ 4 days	CVC	Male gender
		LOS ≥ 7 days			Immobility >72 h	Asymmetric extremity
		OCP			Infection	CVC
		CVC			Orthopedic surgery	Active cancer
		Bacteremia			Major trauma (ISS > 15)	Alternative diagnosis^b^
		Direct ICU admit			Malignancy	
					OCP	
					Burns >30% BSA	
					Thrombophilia	
					Age <1 or >14 years	
					Obesity	
					Hypercoagulable state	

*ICU, intensive care unit; MV, mechanical ventilation; LOS, length of stay; OCP, oral contraceptive pill; CVC, central venous catheters; ISS, injury severity score; BSA, body surface area*.

*^a^Included venous and arterial thromboembolism in their study*.

*^b^Presence of this factor results in point reduction from score*.

Two separate risk-assessment models, one for critically ill children and another for non-critically ill children, were created from a single institution during the same time period by retrospective, case–control study designs (23, 49). In non-critically ill children, scores of 8, 7, and ≤6 correlated to risk of HA-VTE of 12.5, 1.1, and 0.1%, respectively (49). In critically ill children, scores of 15, 7–14, and ≤6 correlated to risk of HA-VTE of 8.8, 1.3, and 0.03%, respectively (23).

Kerlin et al. derived an equation that uses 0 or 1 for absence or presence of certain factors (53):

$$\begin{array}{l} \text{VET probability=\{e\textasciicircum{}[1.086 (male)+0.595 (asymmetric extremity)}\\ \text{ +0.643 (CVC)+0.549 (active cancer)}-\text{1.11 (alternative diagnosis)}\\ -\text{2.03]\}/\{1+e\textasciicircum{}[1.086 (male)+0.595 (asymmetric extremity)+0.643}\\ \text{ (CVC)+0.549 (active cancer)}-\text{1.11 (alternative diagnosis)}-2.03]\} \end{array}$$

Reiter et al. created an ICU-specific risk-assessment model (excluded cardiac ICU) for “pre-hospital” and “in-hospital” thromboses but included both venous and arterial events (52). They derived 12 risk factors from existing literature and weighted them equally with 1 point. They found for every 1 point increase in total score, the risk of a symptomatic thrombosis increased by 1.57-fold (95% confidence interval 0.132–5.49) to 2.12-fold (95% confidence interval 0.175–18.34) for “pre-hospital” and “in-hospital” thrombi, respectively (*p* < 0.05) (52).

The model by Prentiss describes levels of risk, Risk Score 1–3, but neither details which risk factors comprise the scoring system nor their individual point values (51). Additional models have been derived for children with acute lymphoblastic leukemia (54), pediatric oncology patients with a CVC-related VTE (55), and pediatric trauma (56).

To date, a multi-institutional risk-assessment model has been lacking in the literature, but current research is underway to address this gap (57). The Children’s Hospital-Acquired Thrombosis (CHAT) Registry is a multi-institution registry with three planned phases of research. The first phase is a retrospectively derived risk-assessment model based on logistic regression. There are seven institutions contributing cases and controls (1:2), and as of January 1, 2017, there are 647 unique subjects with VTE in the registry (enrollment ongoing). Once the risk-assessment model is developed, it will be prospectively validated it in a separate multi-institutional study, i.e., phase 2. If validated, a randomized controlled trial investigating the utility of the CHAT risk-assessment model will be designed and conducted as the third phase.

## Morbidity

Acute VTE sequelae depend on location and severity. Pulmonary thromboses may lead to pulmonary hypertension or cardiovascular instability. Thrombosis of the superior vena cava (SVC) or thoracic vessels may result in SVC syndrome. Extremity DVTs induce pain and swelling. VTE in patients with renal disease has been associated with 2-fold increase in hospital admissions and 10-fold increase in LOS (53). Neonates have increased risk of chronic renal insufficiency following renal vein thrombosis.

Risk of VTE recurrence has been variably reported but is estimated around 10% in children and 3% in neonates (9, 18). Comorbidities such as cardiovascular disease, malignancy, and neurovascular disorders have been associated with increased likelihood of recurrence. Patients with identified thrombophilia variants, e.g., prothrombin gene mutation and antithrombin deficiency, may also be at increased risk of recurrent VTE. PTS, chronic venous insufficiency associated extremity pain, edema, and stasis dermatitis, appear less frequent and severe in children (12.4%) compared to adults (30%); however, this likely represents underreporting as outcomes studies are limited and most have relatively short durations of follow-up (58). PTS occurs most commonly in the lower extremities. Recently, the Scientific and Standardization Committee of the International Society on Thrombosis and Haemostasis recommended use of a pediatric modification of the Villalta score for adult PTS to standardize reporting in children (11).

## Mortality

Venous thromboembolism in children is associated with increased mortality. VTE mortality rates of 11.4 per 1,000 child-years have been reported, greater than age-specific mortality estimated at 6.4 per 1,000 child-years in a population-based study conducted in Canada (5). This finding is consistent with data from several registries that report all-cause mortality in those with VTE of 9–17%. In one study, over 20% of deaths were noted to occur within 30 days of VTE diagnosis (8). The highest mortality rate is in the youngest patients. Fortunately, mortality rates directly attributed to VTE are low, 1.5–2.2%. Risk of fatality associated with pulmonary embolism is less in pediatrics compared to adults (59).

## Conclusion

Pediatric VTE is increasing in incidence. Key contrasting points between adults and children are that pediatric VTE is more likely to be diagnosed in a hospital and have recognizable antecedent provocation. As incidence increases, associated morbidities, mortality, and health-care costs do likewise. Clearer understanding of pediatric-specific risk factors and validated risk-assessment models are needed to reduce preventable harm and investigate the efficacy of targeted prophylaxis interventions. A multitude of risk factors have been identified for pediatric VTE, but many need further elucidation. Similarly, risk-assessment models to date provide an initial approach but ultimately lack prospective validation and correlation to outcomes. The multi-institution CHAT registry is poised to overcome these limitations and provide details regarding the magnitude of risk attributable to patient, disease, and interventional factors. Initial results are anticipated in 2017. If successful, the CHAT Registry may serve as the foundation of a risk-stratified, randomized controlled trial to evaluate thromboprophylaxis measures in pediatrics.

## Author Contributions

AM and SC cowrote the manuscript, and each revised and edited the entire manuscript.

## Conflict of Interest Statement

The authors declare that the research was conducted in the absence of any commercial or financial relationships that could be construed as a potential conflict of interest.
